# Middle Ear and Mastoid Diseases: Literature Review and New Classification Proposal

**DOI:** 10.7759/cureus.12317

**Published:** 2020-12-27

**Authors:** Fulvio Mammarella, Antonella Loperfido, Melissa Zelli, Claudio Maria Pianura, Gianluca Bellocchi

**Affiliations:** 1 Department of Otolaryngology, San Camillo Forlanini Hospital, Rome, ITA

**Keywords:** cholesteatoma surgery, cholesteatoma and treatment modality, middle ear disease, middle ear surgery, temporal bone surgery

## Abstract

There are many classifications for cholesteatomatous otitis media but none correspond exactly to the formulation of a surgical protocol for the middle ear and mastoid nor are any specific for the extension of the otitis media, making it difficult to interpret the published results.

All the available classifications are limited to the cholesteatoma and, moreover, the variety of the reported studies in literature does not allow a standardization to define middle ear pathology and surgical procedures.

The aim of this paper is to propose a novel staging system for middle ear diseases paying attention to the involvement of tympanic Cavity (C), Attic (A), and Mastoid (M): the CAM classification.

In particular, CAM classification is composed of three symbols (0 / + / ρ) and two letters (t and m, tiny and ivory mastoid cases) related to the alphabetic initial of the macroscopic region, being able to allow a clear and specific description of the local situation. Moreover, the possibility to describe the temporal bone and the potential use of sub-groups to specifically define the involvement of the tympanic cavity and the attic could allow the creation of a common otologic language among all the different centres.

## Introduction and background

The first approaches to the surgical management of middle ear and mastoid diseases date back to the time of the Egyptians. These initial procedures were performed with variable success until the mid-1700s when Jean Petit introduced some innovations and subsequently they were replaced by new techniques proposed by Wilde, Schwartze, and von Troltsch in the 20th century [1].

The great variety of surgical approaches available is due to the ear anatomy and its complexity, the small size of mastoid, the complex spatial relations of important structures, and the possible presence of specific otologic diseases characterized by heterogeneous extension with potential damage.

Therefore, several modern surgical approaches have been codified, especially endoscope-assisted surgical approaches in recent times. However, some questions in modern otology remain open: the necessity of a common language to classify the pre- and post-operative status of otological diseases that can allow a comparison between procedures and their demolitive and functional results. Today, however, there is no standardized staging for the type of surgical procedure and the classification of the extension of the otitis media, making it difficult to interpret the published results. 

The aim of this paper is to provide a review of the literature and to propose a novel classification for middle ear diseases paying attention to the involvement of tympanic Cavity (C), Attic (A), and Mastoid (M): the CAM classification.

## Review

A literature search was conducted to identify all the potential relevant papers about this topic. The main scientific databases used were PubMed, Scopus, and Cochrane Library; the terms used for the search were "middle ear disease", "middle ear surgery" and "cholesteatoma". We considered only papers written in English with no time restriction (all the databases used from their inception until September 2020). Additionally, a manual search of conference papers, book chapters, and academic pamphlets was performed.

Numerous attempts of otological classification have been published in the literature. The first writing dates back to 1964 by the Committee of preservation of hearing of the American Academy of Ophthalmology and Otorhinolaryngology and is a broad classification including all diseases of the middle ear and mastoid to surgical indication; in this work the cholesteatoma was classified as a sub-category [2]. The subsequent classifications were instead characterized by a common denominator: the attempt to create a staging system dedicated to the cholesteatoma. Its genesis is a consequence of the need for a simple and practical staging that allows to standardize the therapy protocols and to compare the results. Austin in 1985 was the first to propose a classification of the cholesteatoma, staging the pathology in four groups [3].

One year later, Meyerhoff proposed a classification based on multiple criteria, as pathophysiology, localization, tubal function, ossicular defects, and presence/absence of any complications. Moreover, he kept attention on the aetiology of the cholesteatomas, differentiating them into primary, secondary, tertiary, and congenital ones [4].

Lau et al. in 1988 first proposed an otoscopic classification that divided the cholesteatoma, based on the localization, into four groups and subsequently revised and simplified the classification defining only three classes. The great limit of these classifications was the failure to describe the mastoid involvement because the authors were not able to visualize this region using the otoscope [5].

This limit was solved by Sanna and Zini in 1993, when they classified five different types of petrous bone cholesteatomas on the basis of the mastoid portion involved: supralabyrinthine, infralabyrinthine, massive labyrinthine, infralabyrinthine-apical, and apical cholesteatoma [6].

Saleh and Milles in 1999 proposed a staging system of cholesteatoma based on the extent of lesion, the ossicular status, and the preoperative complications. This system describes the extent of disease defining the site and the stadium, based on the number of sites involved by pathology. Seven sites are included: the attic, antrum, middle ear, mastoid eustachian tube, labyrinth, and middle fossa. The condition of the ossicular chain was divided into four stages with the functional implications and the possibilities for ossicular reconstruction. Regarding preoperative complications, five different clinical entities were reported: the fistula of the lateral semicircular canal, the facial paralysis, the anacusis, the thrombosis of the breast, and the intracranial extension. This allowed classification of all the lesions except those of the petrous apex, usually rare [7].

Borgstein in 2007 focused his classification to describe the retraction pocket, a typical anatomic aspect associated to cholesteatoma, and he reported specifically his experience on paediatric patients [8]. Telmesani in 2009 proposed the first classification of this pathology combining the clinical criteria with the radiological features described on CT [9].

In 2010 Shin et al. were the authors of the first classification dedicated only to cholesteatomas of the external auditory canal. They distinguished spontaneous cholesteatomas, in the absence of identifiable causes and without stenosis of the external auditory canal, congenital cholesteatomas, in presence of duct stenosis, post-traumatic cholesteatomas, post-inflammatory cholesteatomas and tumour forms. They described the importance of the clinical aspect combined with the imaging on CT to define the location of the pathology and the surgical treatment. The most common wall of the external auditory canal (EAC) involved by the pathology is the posterior one [10].

In 2012 Belal et al. proposed a review of the techniques used in the surgical management of the cholesteatoma of the middle ear and of the mastoid designing a staging system for tympanomastoid cholesteatoma from stage 1 to stage 5 based on the site of disease in the tympanic cavity (T), the mastoid cavity (M), and the presence of any complication (C). To stage any case they performed a clinical (otoscopic/microscopic/endoscopic) examination, a radiological study through the high-definition CT petrous bone and the clinical and radiological correlation. They rejected to describe the potential involvement of the ossicular chain because this is often overestimated and is not a reliable factor because the final status of the ossicular chain cannot be assessed except after the complete eradication of the disease [11].

Presutti and Marchioni in 2015 differentiated epitympanic cholesteatoma from meso-tympanic cholesteatoma, describing further pathological aspects as the features of the lesion, the patient age, and the eustachian tube status [12].

The Japan Otological Society (JOS) in 2017 proposed an interesting and well-structured staging and classification system for middle ear cholesteatoma. In order to represent the extent of the cholesteatoma, they propose the PTAM system, splitting the tympanomastoid space into four potentially involved sections - the protympanum (P), the tympanic cavity (T), the attic (A), and the mastoid (M). Furthermore, they describe a specific staging system for respective cholesteatoma types: pars flaccida cholesteatoma; pars tensa cholesteatoma; cholesteatoma secondary to a tensa perforation; congenital cholesteatoma. They also focus their attention on the development of mastoid cells and on the pathological status of the stapes [13].

The classification used by Potsic should be considered separately because it is probably the only classification of congenital cholesteatoma in pediatric population widely recognized in Western countries, with the exception of Japan where the main reference is the JOS classification, already mentioned [14].

In the literature several types of surgical procedures are described, including a large group of techniques that in some cases might be associated with each other [15]. However, all the mentioned classifications are limited to the cholesteatoma and, moreover, the variety of the reported studies in literature does not allow a standardization to define the medium ear pathology and the surgical procedures. Table 1 reports the limits of the mentioned classifications.

**Table 1:** Limits of the mentioned classifications

Author	Point of weakness
Meyerhoff et al. [4]	Absence of clinical relevance linked to the need for multiple factors to be investigated, some of which are complex in preoperatory phase
Lau et al. [5]	Incompleteness, due to the absence of criteria related to mastoid involvement
Saleh et al. [7]	Complexity, due to the big number of variables
Borgstein et al. [8]	Too much sectorial classification, because of the analysis of the only retraction pocket and limited to the paediatric population
Telmesani et al. [9]	No immediacy
Shin et al. [10]	Limited to the analysis of the only external auditory canal cholesteatomas
Belal et al. [11]	Complexity due to the big number of variables

The aim of the CAM system is to overcome these potential lacks, trying to apply this standardized system to classify most middle ear and mastoid pathologies and not only the cholesteatoma. Furthermore, this proposal helps to define the status of pathology both pre- and post-operative. Any request to document greater complexity is possible with the use of specific supplementary sub-classifications.

**Figure 1:**
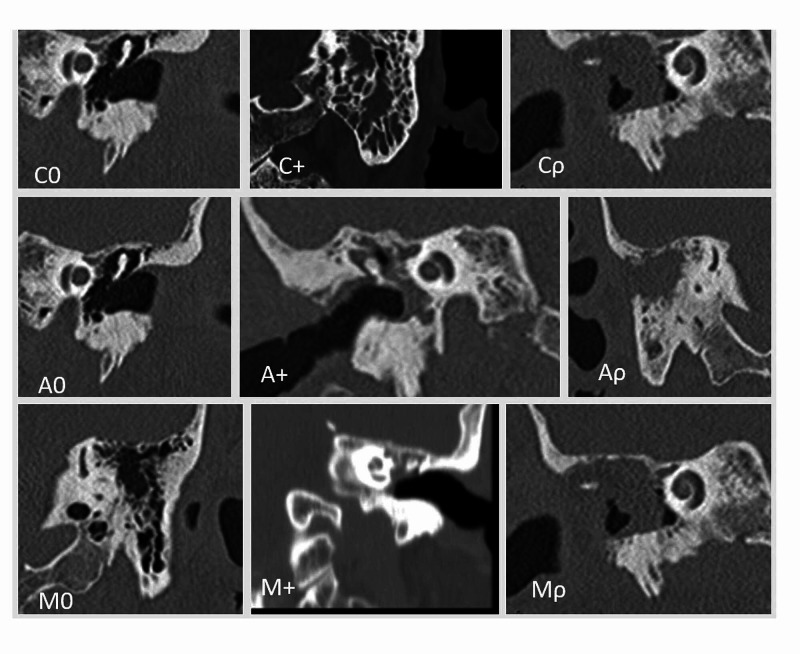
CAM radiological classification

Each individual pathological picture is identified with a symbolic alpha code that summarizes synthetically the localization (tympanic cavity, attic, mastoid) and the main macroscopic characteristics of the pathology. The definition of this pathological picture is the result of multiple pieces of information: anatomical (otoscopy, micro-otoscopy), audiology (audiometry), and radiological (CT) as illustrated in detail in Table 2 and the relative radiological Figure 1.

**Table 2:** CAM staging system

C0	Tympanic cavity without evidence of disease
C+	Opacification of tympanic cavity
Cρ	Tympanic cavity with radiological sign of erosion (ossicular chain, posterior wall)
Ct	Tympanic cavity without evidence of disease and conductive hearing loss (audiometry)
A0	Attic without evidence of disease
A+	Opacification of the attic
Aρ	Attic with otoscopic and radiological sign of erosion
M0	Mastoid without evidence of disease
M+	Opacification of the mastoid
Mρ	Mastoid with radiological sign of erosion
m	Ivory mastoid

It is necessary to specify the time of the evaluation distinguishing clinical, before surgical procedure (cCAM) and pathological, after surgical procedure (pCAM).

As described, in the basic classification there are additional sub-classifications: 1) Subgroups of the local extension of the cholesteatoma in the tympanic cavity. The subgroups are listed in alphabetical order, which can be associated with each other in any combination. This strategy lets define the specific anatomical sub-sites involved, recalling the tympanic sinus as the most common location of recurrence, as shown in Table 3. The definition of these subgroups follows the revision of the anatomical data described in the listed classifications: Telmesani et al. describe that the most common sites of recurrence are the posterior attic (93.7%) and the posterior tympanic sinus (81.2%) [9]. In the study proposed by Saleh et al., the most common sites involved are the attic and the marginal pars tensa, thus making it necessary to introduce a descriptive topographical criterion [7]; 2) Subgroups of attical opacification/erosion ( A+/Aρ). The subgroups are listed in alphabetical order, which can be associated with each other in any combination. It has been designed primarily for preoperative planning purposes, as described in Table 4; 3) Classification of the extracranial extension of cholesteatoma to main structures (labyrinth, petrous apex, tegmen). It is applicable in addition to the basic classification by affixing the last letter. In case there is only the possibility of erosion, it does not require a symbol. The three additional letters can be combined, respecting the alphabetical order, as shown in Table 5. Intracranial complications have not been described because in these cases the role of the neurosurgeon should prevail.

**Table 3:** Subgroups of the local extension of the cholesteatoma in the tympanic cavity

Subgroups	Tympanic cavity
h	Hypotimpanum
r	Protympanum
r	Retrotympanum

**Table 4:** Subgroup of attical opacification/erosion (A+/Aρ)

Subgroups	Attic
a	Anterior
l	Lateral
m	Middle
p	Posterior

**Table 5:** Classification of the extracranial extension of cholesteatoma to main structures

L	Inner ear with radiological sign of erosion (labyrinthine fistula)
R	Petrous apex with radiological sign of erosion
T	Tegmen with radiological sign of erosion

We propose the use of an alphasymbolic system for classifying the pathology involving the middle ear and the mastoid. With its use, our aim is to realize a simple and micro-detailed system to describe the extension and the entity of disease allowing preoperative planning, the comparison of demolitive and functional results as well as the appropriateness of the technique used. The only pathological entity excluded from this classification is the simple tympanic perforation that does not involve the described districts and foresees as the only type of approach to the myringoplasty (over or under).

In the literature is already described the use of the first letter of the anatomic district involved by pathology, usually associated with a number that indicates the specific local involvement described on CT imaging [16]. The large amount of further new classifications and reworks has been the consequence of the difficulty and the complexity to define an immediate and exhaustive description of the cholesteatoma and its extension. None of the previous classifications has proposed an alphasymbolic system. This method could allow for an immediate and simple way to define the pathology through the use of three simple symbols: 0 for not pathological, + for opacation , ρ (ro) for eroded. The increasing involvement of the middle ear is defined by a progressive numeration: A0-A1-A2-A3.

A fundamental assessment to classify the stadium of pathology is the CT without contrast for the preoperative evaluation. CT scan is certainly the most used method even if it has undergone considerable developments over the years and especially in relapses it is associated with MRI diffusion-weighted imaging (DWI) [17].

Telmesani et al. describe as pathologic radiological criteria the presence of local opacity, particularly in the attic, in the eardrum, in the posterior tympanic sinus, or in the mastoid and the presence of the erosion of the scutum. However, usually the CT study of the middle ear and mastoid is performed when the medium ear is inflamed, not allowing a clear diagnosis. Presutti et al. report a percentage from 20 to 70% of cases of imaging opacification where the CT is not able to distinguish the cholesteatoma by inflammation tissue, granulation, fibrosis, or mucous secretions [16]. So, it could be difficult to diagnose or to exclude the presence and the extension of a cholesteatoma only based on CT imaging. Is interesting to see how some authors decide to not operate despite the local opacity described on ear CT: Aoki reports that in only six cases out of 24 he performed an antrostomy despite a diffuse opacity of middle ear and mastoid [18].

The complexity of pathology in structural and functional terms cannot be macroscopically described only using three symbols ( o / + / ρ ). So, two more letters are included: t, to indicate the presence of a conductive hearing loss in a negative CT, and m, tiny, to point out the possible condition of an ivory mastoid. The combination of these features allows a fast and easy comprehension of the anatomic structures and the local involvement of cholesteatoma. With the aim to define a specific otologic diagnostic tool, two more under-classifications (respectively for the tympanic cavity and the attic) and one more for the involvement of neighbouring districts give additional anatomical information. About the status of the chain ossicles, it is described only in case of macroscopic erosion, because of the high risk to overestimate the pathological involvement of ossicles. The CAM classification describes the subarea involved by pathology using the first letter. For a cholesteatoma of the attic, the specific part of the attic occupied by the disease is so defined: a-anterior; l-lateral; m-medial; p-posterior. For a cholesteatoma of the cavity, the letter h is used for hypotympanic involvement, p for protympanic, and r for retrotympanic. In the cases of multiple parts involved, the additional nomenclature respects the alphabetical order: a-> l-> m-> p for a cholesteatoma of the attic, h-> p> r for a cholesteatoma of the cavity.

**Figure 2:**
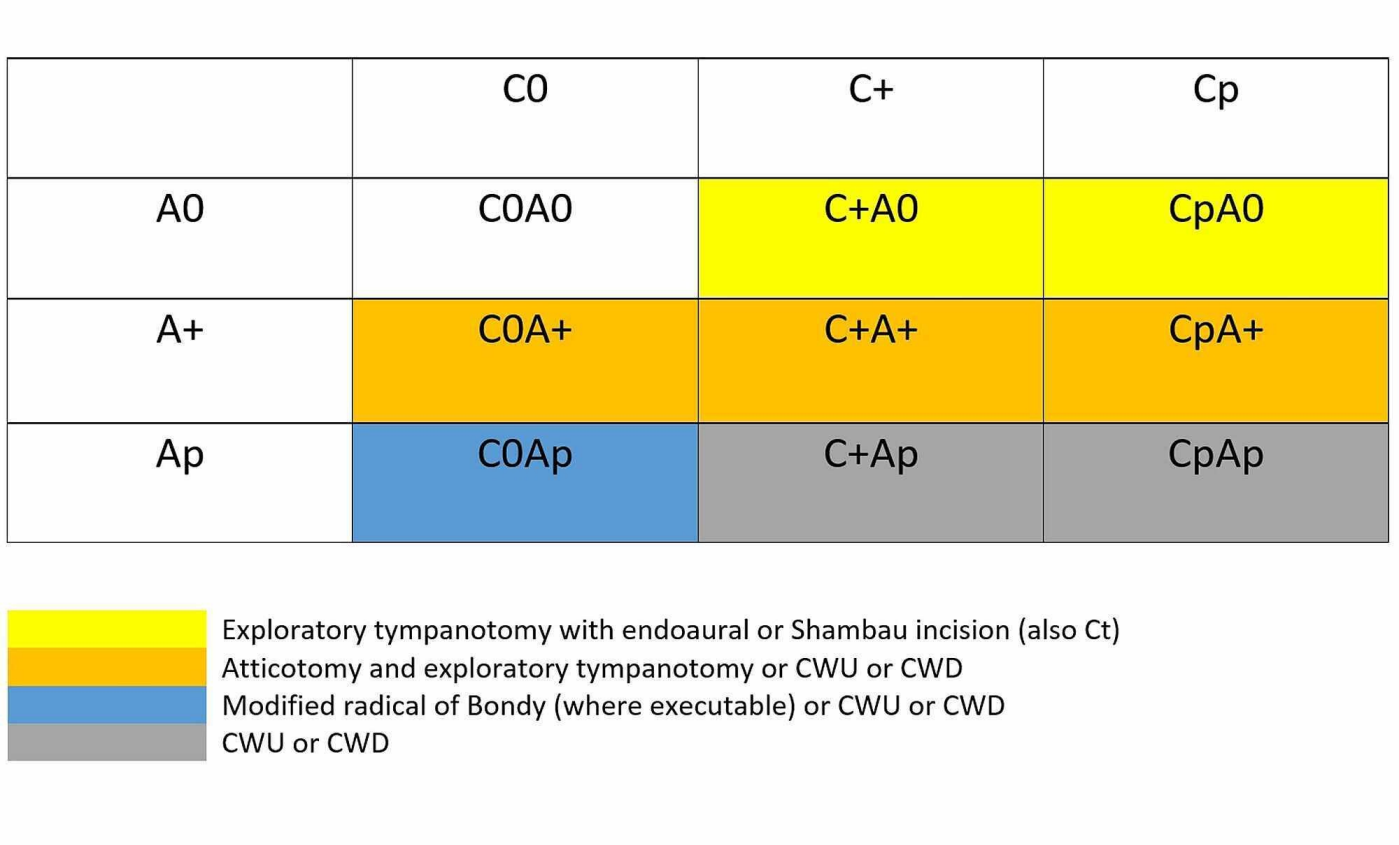
Pre-operative planning the approach type Canal wall down (CWD), canal wall up (CWU)

Furthermore, the CAM classification aims to the possibility of evaluating in the pre-operative planning the approach type as shown in Figure 2.

The aim of this preoperative description of the attic subareas involved by pathology is to define if it should be the eventuality of a surgical ossicular chain dislocation to have a better visualization of the operating field. For example, the involvement of the medial attic portion (A+m) is associated with a poor vision of this region, so it should be necessary to dislocate the ossicles to remove the cholesteatoma. On the opposite, the involvement of the anterior and the posterior districts (A + ap) does not usually imply the use of this procedure. Furthermore, in the cases of involvement of the tympanic sinus, it could be useful to consider the surgical option of a sub-facial tympanostomy (C + r).

CAM classification is composed only of these two subclassifications (A + m / C + r ) with the aim to make this system easy and fast to use in the otologic practice.

## Conclusions

The aim of the CAM classification is to propose a fast and simple way to define pre- and post-operative staging of middle ear and mastoid pathology, describing its entity and extension. In particular, CAM classification is composed by three symbols (0 / + / ρ) and two letters (t and m, tiny and ivory mastoid cases) related to the alphabetic initial of the macroscopic region, being able to allow a clear and specific description of the local situation.

Moreover, the possibility to describe the temporal bone and the potential use of sub-groups to specific define the involvement of the tympanic cavity and the attic, could allow to create a common otologic language among all the different centres.
